# On the descent of the epididymo-testicular unit, cryptorchidism, and prevention of infertility

**DOI:** 10.1186/s12610-017-0065-8

**Published:** 2017-11-14

**Authors:** Faruk Hadziselimovic

**Affiliations:** 1 Cryptorchidism Research Institute, Kindermedizinisches Zentrum Liestal, Liestal, Switzerland; 2 Pediatrics at the University of Basel and Director of Cryptorchidism Research Institfigute, Kindermedizinisches Zentrum, Bahnhofplatz 11, 4410 Liestal, Switzerland

**Keywords:** Epididymo-testicular descent, Cryptorchidism, RNA sequencing, GnRHa-treatment, Mini-puberty, Infertility, Descente épididymo-testiculaire, Cryptorchidie, Séquençage d’ARN, Analogue du Gn-RH, Minipuberté, Infertilité

## Abstract

This comprehensive review provides in-depth coverage of progress made in understanding the molecular mechanisms underlying cryptorchidism, a frequent pathology first described in about 1786 by John Hunter. The first part focuses on the physiology, embryology, and histology of epididymo-testicular descent. In the last 20 years epididymo-testicular descent has become the victim of schematic drawings with an unjustified rejection of valid histological data. This part also includes discussion on the roles of gonadotropin-releasing hormone, fibroblast growth factors, Müllerian inhibiting substance, androgens, inhibin B, and insulin-like 3 in epididymo-testicular descent. The second part addresses the etiology and histology of cryptorchidism as well as the importance of mini-puberty for normal fertility development. A critical view is presented on current clinical guidelines that recommend early orchidopexy alone as the best possible treatment. Finally, by combining classical physiological information and the output of cutting-edge genomics data into a complete picture the importance of hormonal treatment in preventing cryptorchidism-induced infertility is underscored.

## Résumé

Cette revue complète traite en profondeur les progrès réalisés dans la compréhension des mécanismes moléculaires à la base de la cryptorchidie, une pathologie fréquente décrite pour la première fois aux environs de 1786 par John Hunter. La première partie est. centrée sur la physiologie, l’embryologie et l’histologie de la descente épididymo-testiculaire. Durant les 20 dernières années, la descente épididymo-testiculaire est. devenue la victime de dessins schématiques associés à un rejet injustifié de données histologiques valides. Cette partie discute aussi les rôles qu’ont dans la descente épididymo-testiculaire l’hormone libérant les gonadotrophines, les facteurs de croissance fibroblastiques, l’hormone antimüllérienne, les androgènes, l’inhibine B, et l’insuline-like 3.

La seconde partie aborde l’étiologie et l’histologie, ainsi que l’importance de la minipuberté pour un développement normal de la fertilité. Un regard critique est. porté sur les recommandations cliniques actuelles qui conseillent la seule orchidopexie précoce comme le meilleur traitement possible. Finalement, en combinant les informations issues de la physiologie classique et la production des données génomiques les plus en pointe dans un tableau complet, l’importance du traitement hormonal dans la prévention de l’infertilité induite par la cryptorchidie est soulignée.

## Epididymo-testicular descent

Several animal studies have shown that the testis regulates its own descent by secreting hormones [for a summary see [1]. Furthermore, it is generally believed that hormonal stimulation induces a gubernacular reaction that is thought to be the most important mechanism for successful descent of the male gonad [1–4]. That said, complete early neonatal transection of the gubernaculum does not prevent epididymo-testicular descent [5] calling its postulated role into question.

**Fig. 1:**
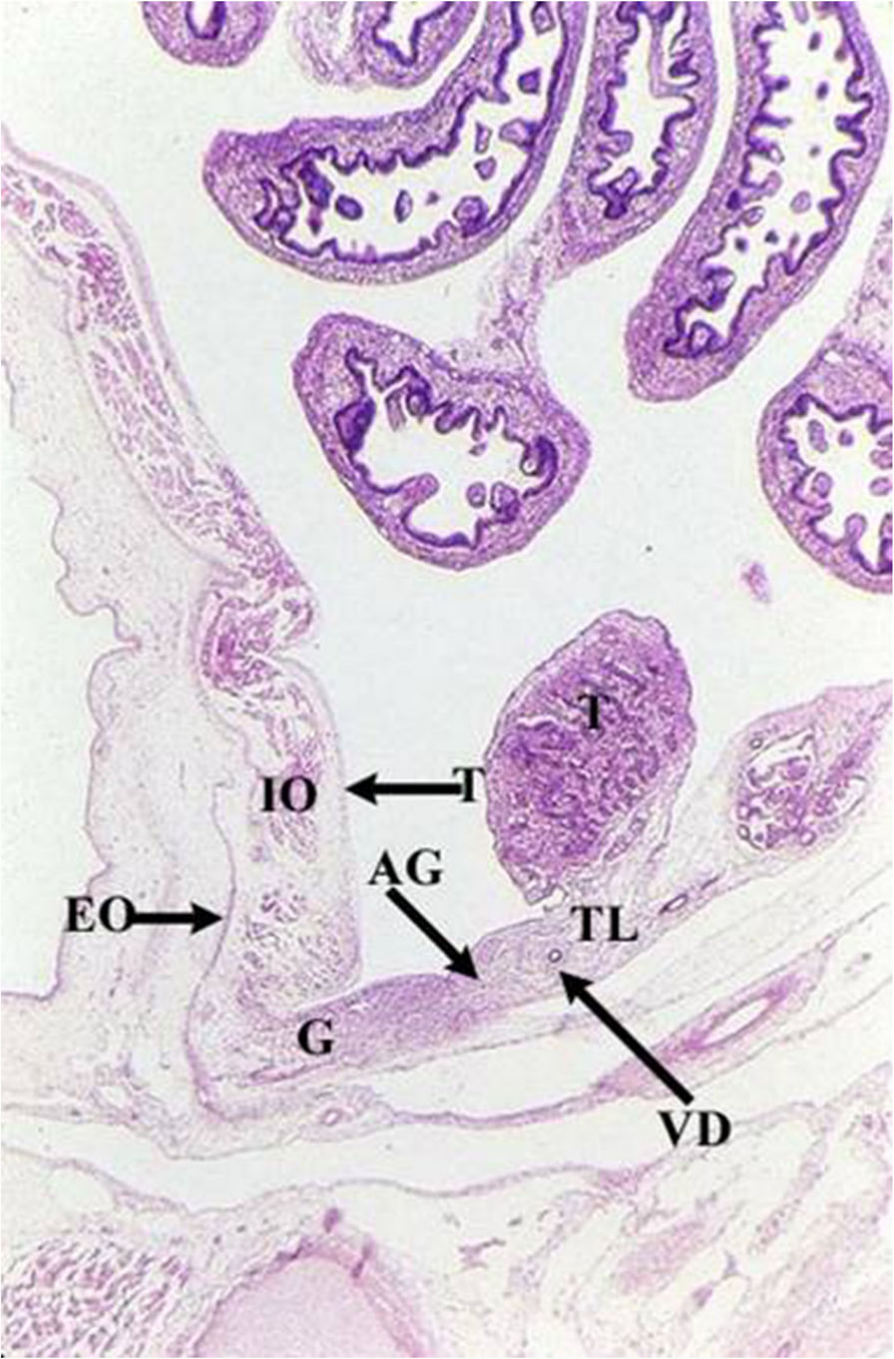
10-week-old male fetus with testis (T) located intra-abdominally and intraperitoneally. Testis is connected to Wolffian duct (VD) with the testicular ligament (TL) while the gubernaculum, which is divided into two parts, inserts proximally into the Wolffian duct via an intraabdominal part (AG) and an inguinal part (G). Internal abdominal oblique muscle (IO) and fascia of extra-abdominal oblique muscle (EO and arrow) as well as fascia from transversal abdominal muscle (T and arrow) are labeled

**Fig. 2:**
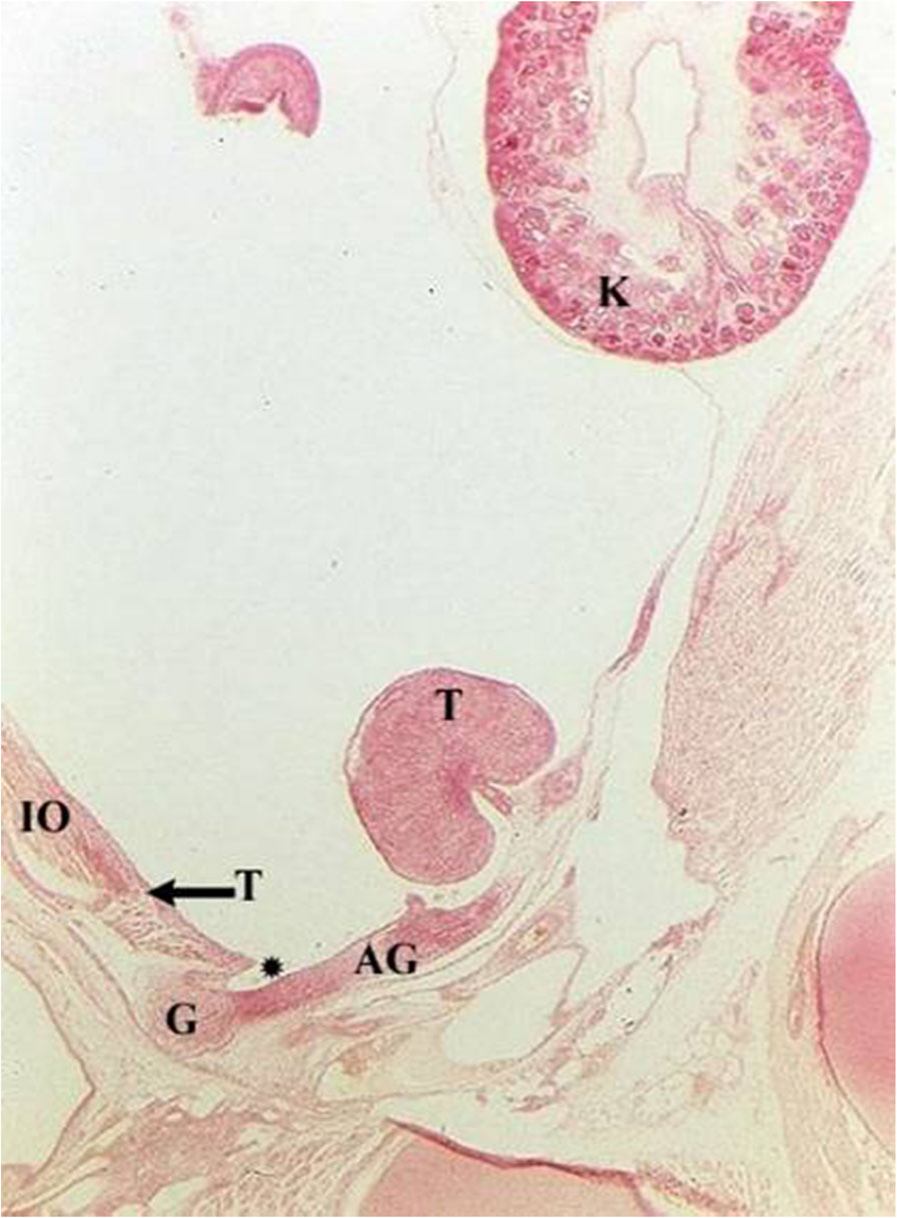
Four-month-old fetus with intra-abdominal and intra-peritoneal testis (T) with meso-testis at the dorsal abdominal wall below the kidney (K). Processus vaginalis starts to develop (*). The topographical relation of gubernaculum and developing Wolffian duct is identical as in Fig. 1. However, in parallel to the new formation of processus vaginalis the inguinal part of the gubernaculum also enlarges

**Fig. 3:**
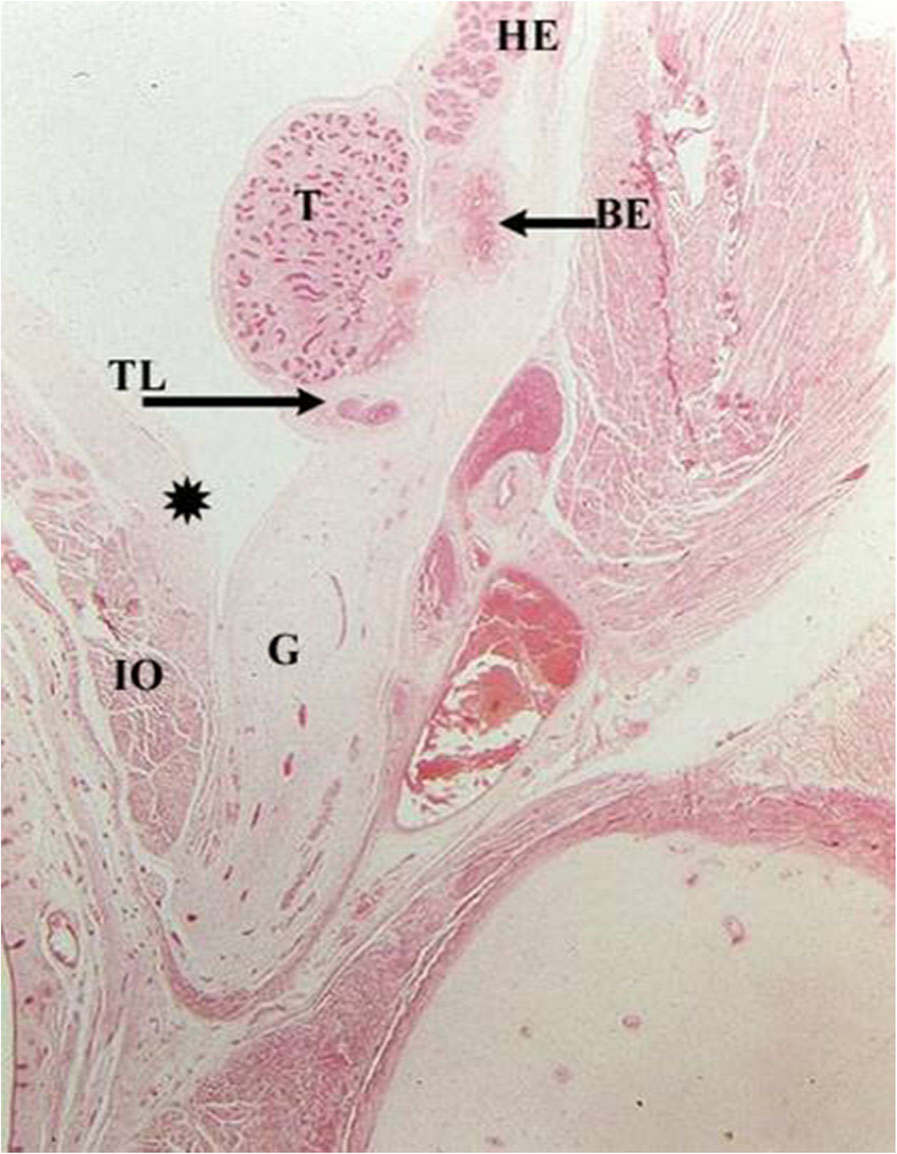
Sagittal cut through epididymo-testicular unit of a five-month-old fetus. Testis (T) is surrounded by head (HE), body (BE) and tail (TL) of the epididymis. The testicular ligament, the connection between testis and the tail of the epididymis (TL) becomes narrow. The inguinal parts of gelatinous gubernaculum (G) show cremaster muscle fibers at its periphery. Processus vaginalis (*) and internal oblique muscle (IO) are indicated

**Fig. 4:**
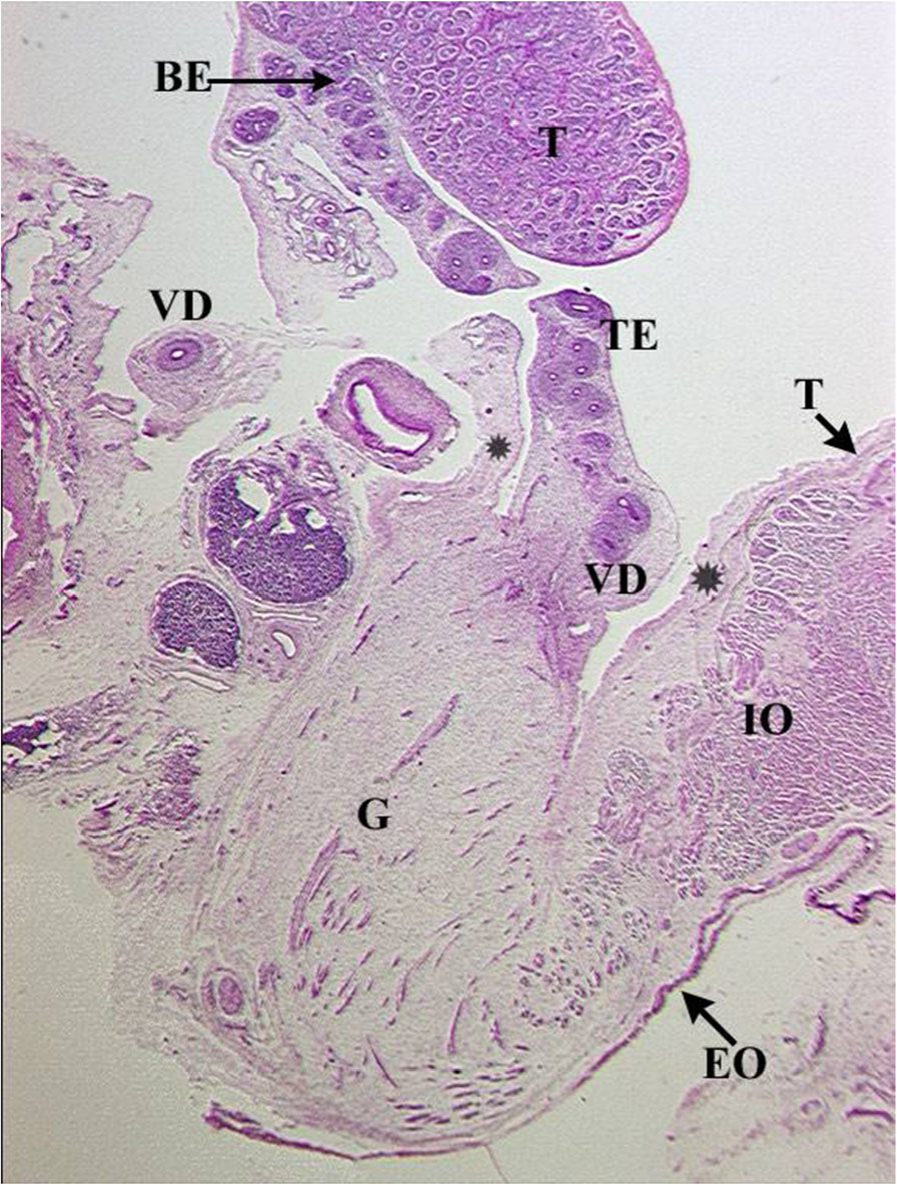
Topography of epididymo-testicular unit presented in serial sagittal sections (from distal to proximal) of 6-month-old fetus. The gubernaculum (G) is a thick cord composed of loose mesenchymal tissue with irregularly arrayed thin cremaster muscle fibers at periphery. At its proximal end, (AG) the gubernaculum is connected to the tail of the epididymis (TE, arrow). Short testicular ligament (TL) is connecting the tail of the epididymis to the testis (T). Notice; gubernaculum is never directly attached to the testis but always to the tail of the epididymis. [Deep inguinal ring and processus vaginalis (asterisk), transversus muscle fascia (T, arrow), internal oblique muscle fascia (IO), external oblique muscle fascia (EO), body of the epididymis (BE), deferent duct (VD)]

**Fig. 5:**
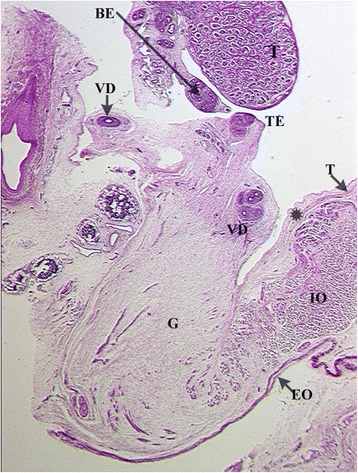
Topography of epididymo-testicular unit presented in serial sagittal sections (from distal to proximal) of 6-month-old fetus. The gubernaculum (G) is a thick cord composed of loose mesenchymal tissue with irregularly arrayed thin cremaster muscle fibers at periphery. At its proximal end, (AG) the gubernaculum is connected to the tail of the epididymis (TE, arrow). Short testicular ligament (TL) is connecting the tail of the epididymis to the testis (T). Notice; gubernaculum is never directly attached to the testis but always to the tail of the epididymis. [Deep inguinal ring and processus vaginalis (asterisk), transversus muscle fascia (T, arrow), internal oblique muscle fascia (IO), external oblique muscle fascia (EO), body of the epididymis (BE), deferent duct (VD)]

**Fig. 6:**
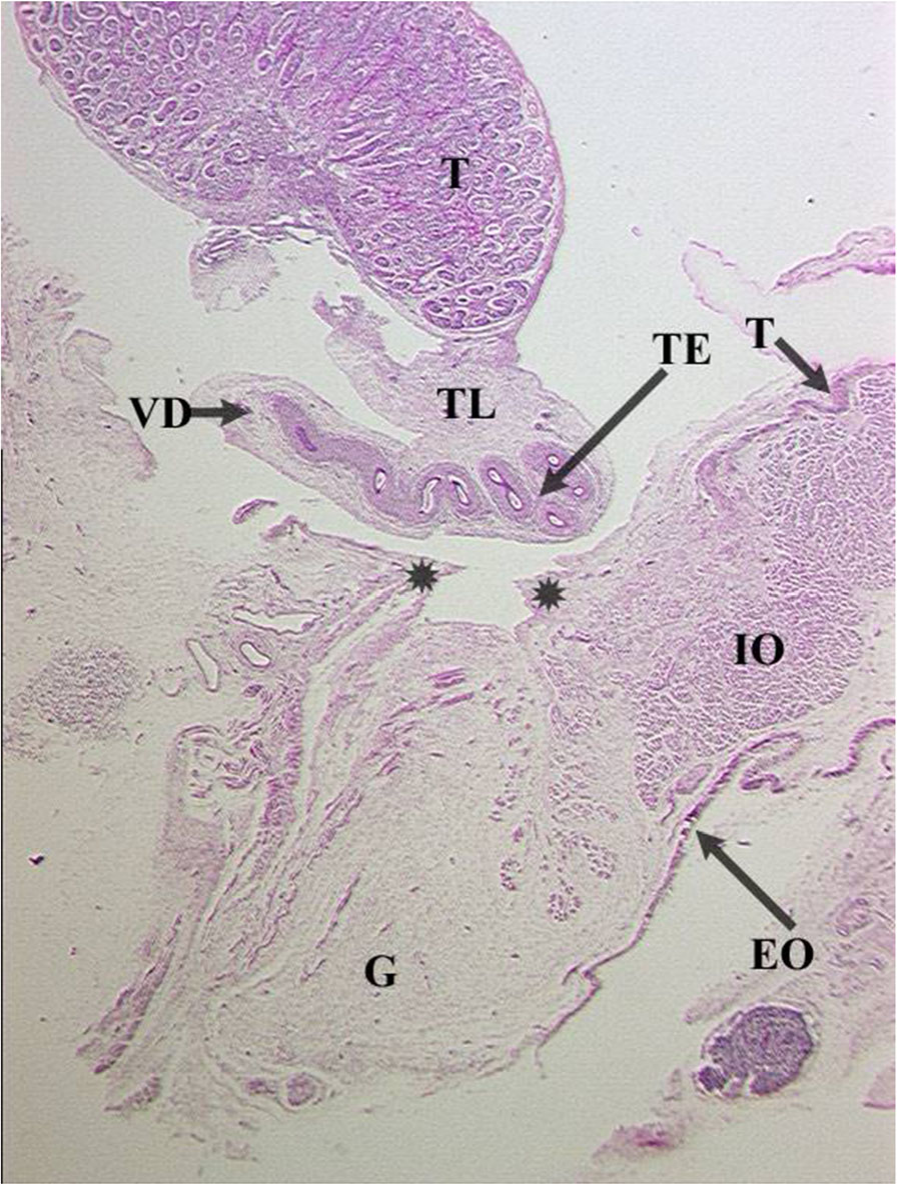
Topography of epididymo-testicular unit presented in serial sagittal sections (from distal to proximal) of 6-month-old fetus. The gubernaculum (G) is a thick cord composed of loose mesenchymal tissue with irregularly arrayed thin cremaster muscle fibers at periphery. At its proximal end, (AG) the gubernaculum is connected to the tail of the epididymis (TE, arrow). Short testicular ligament (TL) is connecting the tail of the epididymis to the testis (T). Notice; gubernaculum is never directly attached to the testis but always to the tail of the epididymis. [Deep inguinal ring and processus vaginalis (asterisk), transversus muscle fascia (T, arrow), internal oblique muscle fascia (IO), external oblique muscle fascia (EO), body of the epididymis (BE), deferent duct (VD)]

**Fig. 7:**
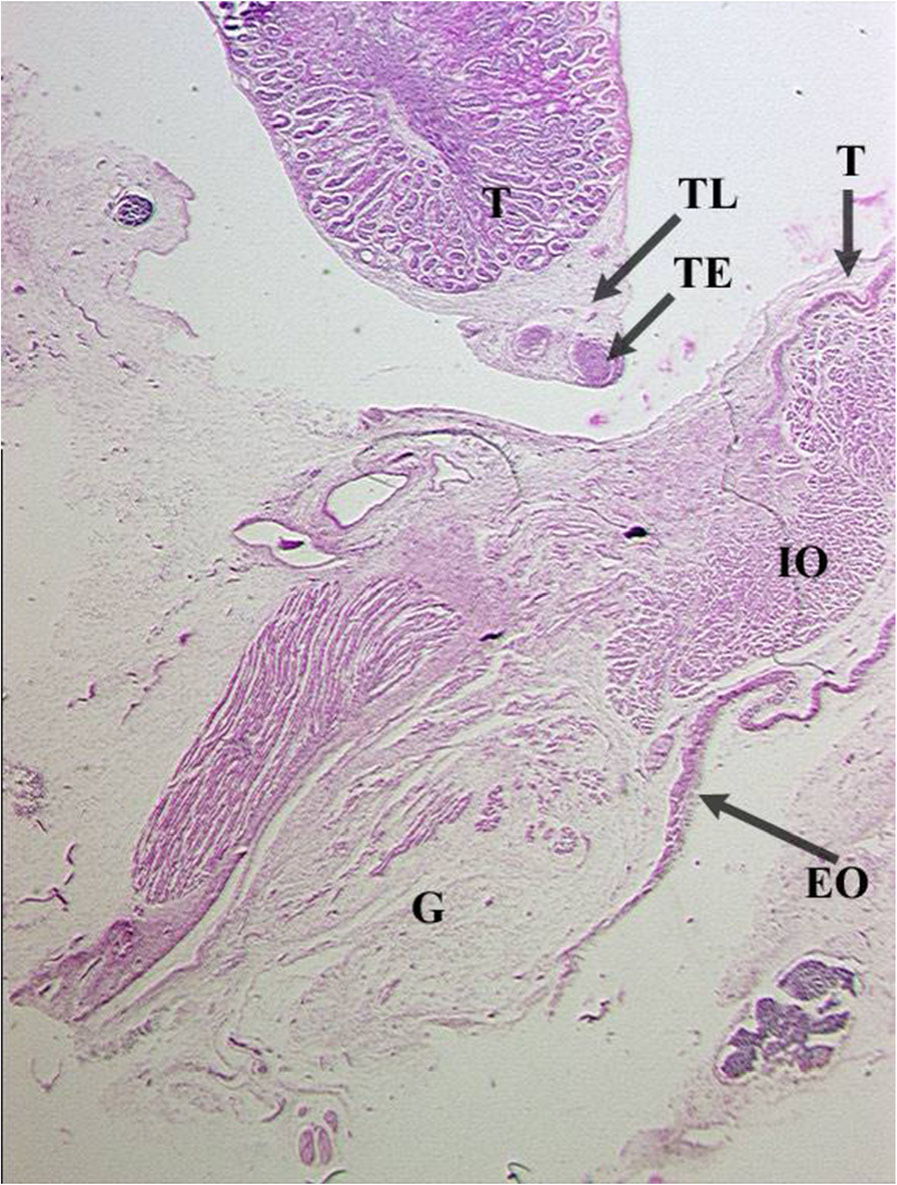
Topography of epididymo-testicular unit presented in serial sagittal sections (from distal to proximal) of 6-month-old fetus. The gubernaculum (G) is a thick cord composed of loose mesenchymal tissue with irregularly arrayed thin cremaster muscle fibers at periphery. At its proximal end, (AG) the gubernaculum is connected to the tail of the epididymis (TE, arrow). Short testicular ligament (TL) is connecting the tail of the epididymis to the testis (T). Notice; gubernaculum is never directly attached to the testis but always to the tail of the epididymis. [Deep inguinal ring and processus vaginalis (asterisk), transversus muscle fascia (T, arrow), internal oblique muscle fascia (IO), external oblique muscle fascia (EO), body of the epididymis (BE), deferent duct (VD)]

**Fig. 8:**
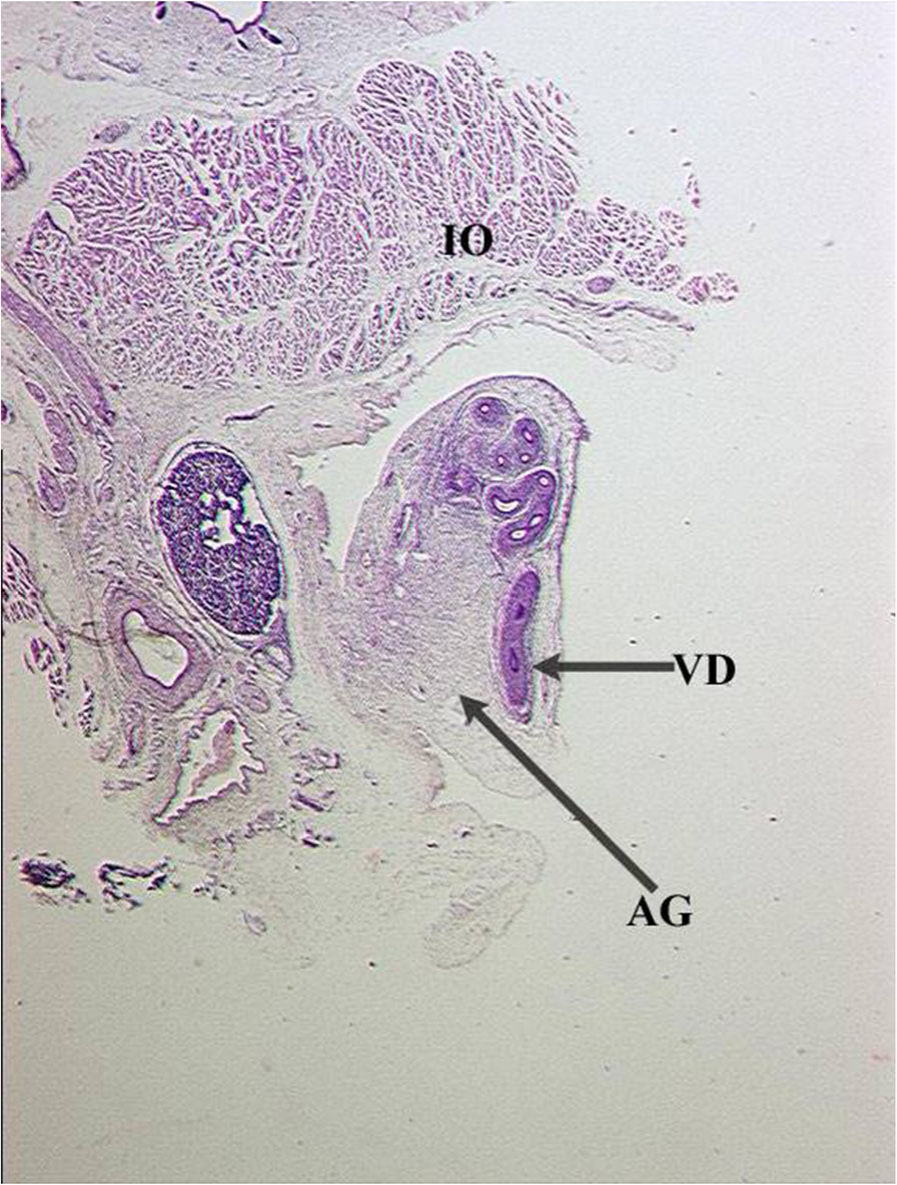
Topography of epididymo-testicular unit presented in serial sagittal sections (from distal to proximal) of 6-month-old fetus. The gubernaculum (G) is a thick cord composed of loose mesenchymal tissue with irregularly arrayed thin cremaster muscle fibers at periphery. At its proximal end, (AG) the gubernaculum is connected to the tail of the epididymis (TE, arrow). Short testicular ligament (TL) is connecting the tail of the epididymis to the testis (T). Notice; gubernaculum is never directly attached to the testis but always to the tail of the epididymis. [Deep inguinal ring and processus vaginalis (asterisk), transversus muscle fascia (T, arrow), internal oblique muscle fascia (IO), external oblique muscle fascia (EO), body of the epididymis (BE), deferent duct (VD)]

**Fig. 9:**
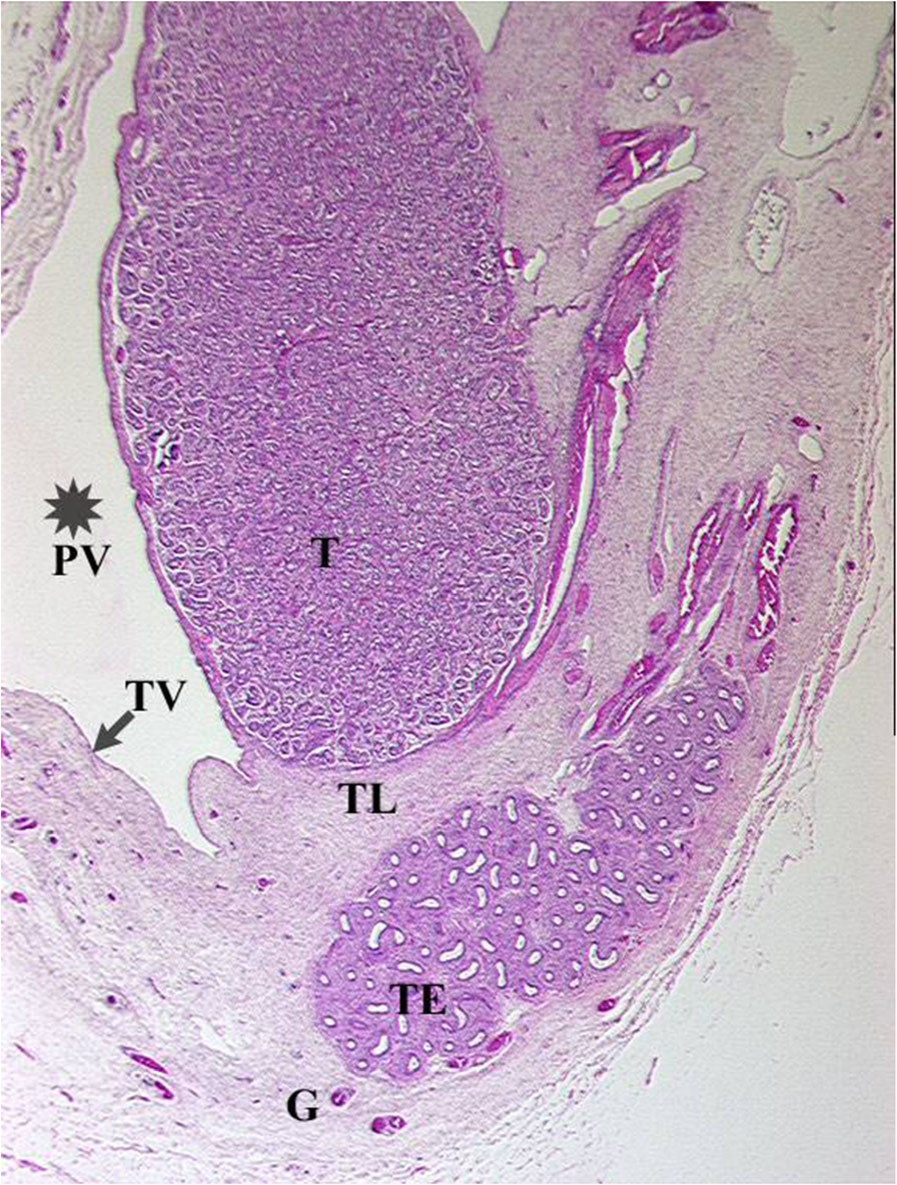
24 weeks old fetus, sagittal sections displaying topographical relations between processus vaginalis, testis, epididymis, ligamentum testis and gubernaculum

**Fig. 10:**
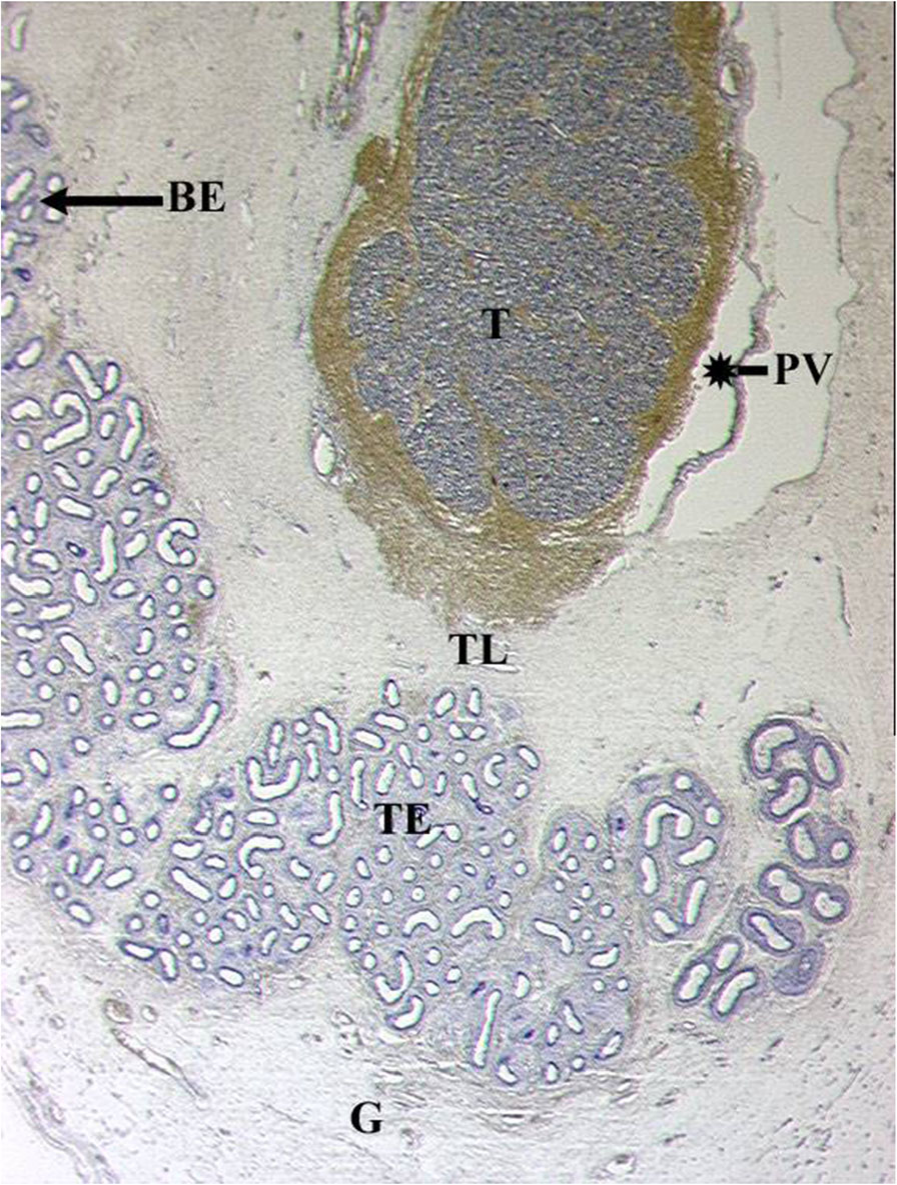
Sagittal section of a newborn’s body and the tail of epididymis and their topographical relation to processus vaginalis (*), testis (T), ligamentum testis and gubernaculum. The gubernaculum is virtually inexistent (G) and the testicular ligament (TL) undergoes the process of retroperitonealization and becomes a part of the scrotal wall. [Histological sections are personal material obtained from Töndery-Collection Institute of Anatomy Zürich]

**Fig. 11:**
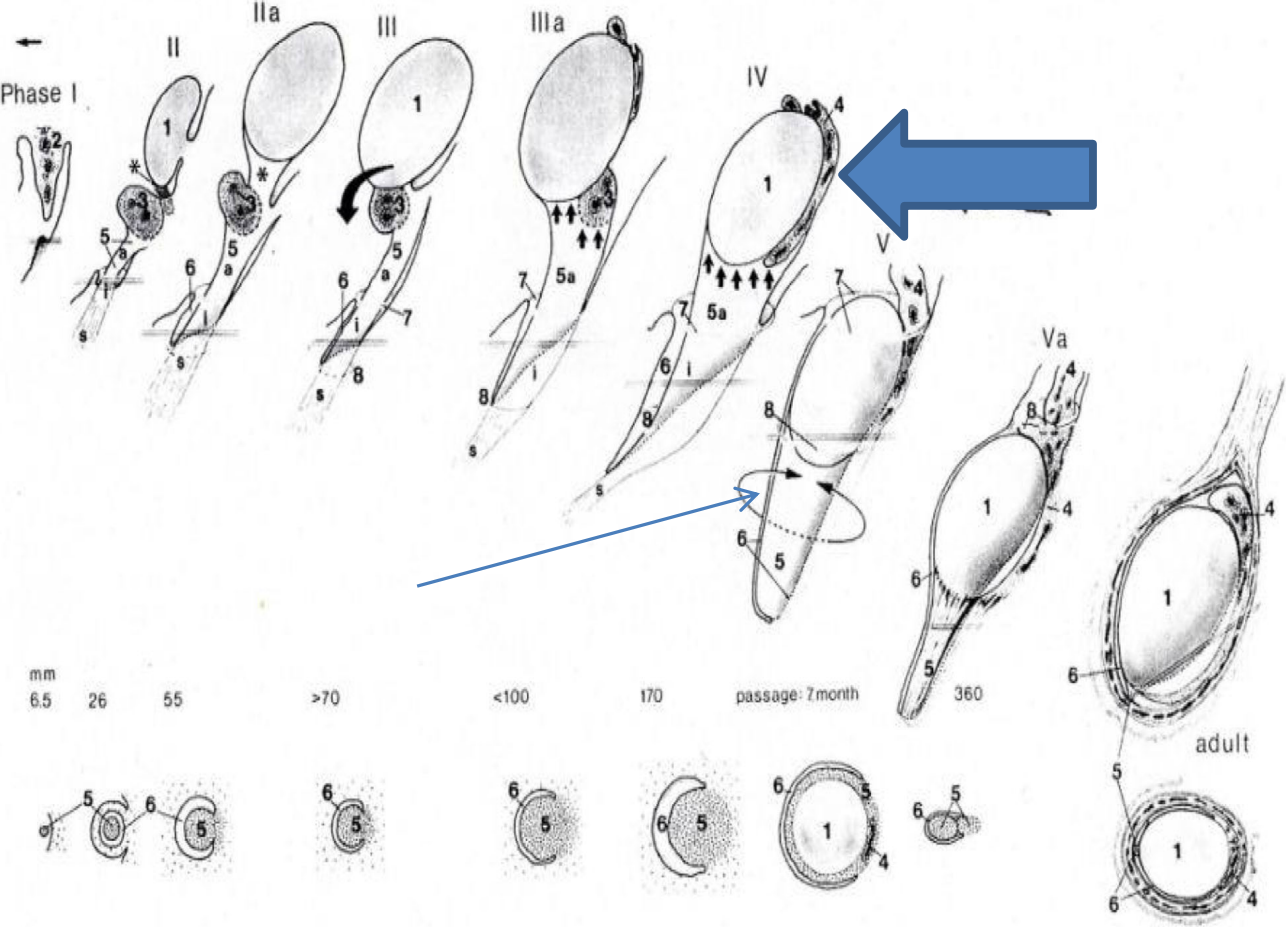
Synopsis of testicular descent from Barteczko and Jakob [3]. Gubernaculum is sketched to be connected to the caudal testicular pole during the entire descent.(arrow). This however could not be confirmed by precise histological pictures. Furthermore, topographical relation of developing epididymis and testis are incorrectly pictured in that epididymis and particular its tail appears small and atrophied (Fat arrow)

Rather than gubernaculum elongation and differentiation, it is actually the developing epididymis that enlarges and holds the testis towards the developing scrotum during the process of descent (Figs. 1, 2, 3, 4, 5, 6, 7, 8, 9 and 10). Histological sections clearly show that the epididymis precedes the testis throughout the entire descent (Figs. 1, 2, 3, 4, 5, 6, 7, 8, 9 and 10). Furthermore, no migration of the gelatinous gubernaculum as presented in a schematic drawing by Hutson is visible in a series of sagittal histological sections [4] (Figs. 4, 5, 6, 7, 8, 9 and 10). In contrast, carried by the epididymis, each testis descends from the dorsal abdominal wall into the scrotum, together with a gelatinous gubernacular mass, that dilates the inguinal canal and thus creates space for the descent of the epididymo-testicular unit [6]. In a graphic drawn synopsis of testicular descent, Barteczko and Jakob connected the gubernaculum to the caudal testicular pole during the entire descent [3] (Fig. 11). This depiction however, is not confirmed by precise histological examination and therefore needs to be corrected accordingly (Figs. 1, 2, 3, 4, 5, 6, 7, 8, 9, 10 and 11). The gubernaculum is attached proximally either to the Wolffian duct or to the caudal pole of the epididymis, but never to the testis (Figs. 1, 2, 3, 4, 5, 6, 7, 8, 9 and 10).

Evidence so far suggests that Wolffian duct elongation occurs primarily through a combination of cell proliferation and cell rearrangements [7]. In rodents Wolffian duct coiling moves from proximal to distal in a temporal fashion and the initial stages are planar (i.e., the coiling occurs at the two-dimensional level) [7]. It has been hypothesized that the reason for the length of the duct is that without it sperm maturation would not have enough time to occur [7]. In turn, coiling of the Wolffian duct into a 6 m long ductus epididymis that is then packed into a 5-cm long epididymal sheet starting from the proximal towards the distal produces forces that are important for epididymo-testicular descent [8]. Thus, disturbances of epididymal development results in a cryptorchid position of the epididymo-testicular unit [6, 9, 10].

### The roles of gonadotropin-releasing hormone (GnRH) and fibroblast growth factors (FGFs)

GnRH neurons are essential for epididymo-testicular descent and the onset and maintenance of sexual reproduction. Proper specification of GnRH cell fate, both prenatally and postnatally, relies on FGFs [11], and the developing GnRH system is highly sensitive to reduced levels of FGF signaling [12]. FGF-mediated signaling is involved in mitogenesis, proliferation, differentiation, cellular migration, angiogenesis, and tissue injury repair [13]. *FGFR1* hypomorphy severely reduces the total number of GnRH neurons, inducing congenital hypogonadotropic hypogonadism, either alone or in association with other hypothalamic-pituitary deficiencies [12]. Severe hypoplastic crypto-epididymis has been observed in transgenic mice with migratory arrest of GnRH neurons [14], as well as in hypogonadal mice lacking GnRH [15], and in mice with loss-of-function mutations in the *GnRH* receptor gene [16], which underlines the importance of GnRH in this developmental process. Hypogonadal male mice lacking *GnRH* are cryptorchid but have a normal gubernaculum, and gonadotropin treatment leads to normal testes development and descent [15].

In cryptorchid boys, GnRH treatment reportedly induces increased testosterone secretion and stimulates further epididymis development and completion of epididymo-testicular descent [17]. Boys with successful descent of the epididymis and testis have a normal-sized epididymis, while the majority of non-responders to hormonal treatment have a small and underdeveloped epididymis [17]. Of interest, luteinizing hormone (LH) receptor knockout mice exhibit bilateral cryptorchidism that can be corrected by testosterone replacement therapy [18]. Specifically, this therapy reverses all of the morphological and gene expression changes in the knockout mice, except those related to insulin-like factor3 (*Insl3*), suggesting that testosterone rather than INSL3 facilitates completion of testicular descent [18]. Furthermore, in 66% of naturally cryptorchid mice, treatment with LH-RH hormone reportedly induces epididymo-testicular descent, while increasing testosterone secretion and normalizing the morphology of an underdeveloped cryptorchid epididymis [19].

### Androgens and *FGFR1*

Androgens are the primary factors regulating epididymal development and function. However, a large body of evidence suggests that fibroblast growth factors also play important roles in the regulation and maintenance of the epididymis. In recent years, it has become clear that FGF signaling is involved in the development and normal functioning of male reproductive organs, such as the testis and epididymis [20]. For example, *Fgf10* is expressed in the mesenchyme of the lower Wolffian ducts and regulates epithelial growth of the seminal vesicles and prostate [21]. In addition testosterone treatment increases “andromedins” *Fgf10* transcription in the seminal vesicles [22] and at later stages of epididymal development; *FGFR1* is specifically expressed in the undifferentiated mesenchyme [20, 23].

Moreover, *FGFR1* mutations have been described in cases of idiopathic hypogonadotropic hypogonadism and cryptorchidism [24, 25]. In 2010, we reported impaired *FGFR1* expression in the undescended testis but not in the descended gonad of unilateral cryptorchid boys [26]. Additionally, decreased FGFR1 protein levels have been found in cryptorchid epididymides of both humans and rodents [10]. These findings support the involvement of FGFR1 in regulating epididymal mesenchyme development [10]. It appears likely that the impaired FGFR1 protein secretion found in underdeveloped mesenchyme in cryptorchid humans and rodents contributes to defective epididymis formation and the subsequent undescended position. Based on our results, a subtle dysfunction (expression) of *FGFR1, SOS1* and *RAF1* is possibly involved in the development of unilateral or bilateral cryptorchidism. Many cases of syndromic crypto-epididymis, as well as a majority of isolated cases, have in common either a disturbance of *FGFs, FGFR1, FGFR2, FGFR3,* and/or a disturbance of the genes involved in regulating the hypothalamic-pituitary-gonadal axis [10]. Normally, muscle development requires signaling by members of the FGF family and their downstream effector early growth receptor 1 (*EGR1)* [27]*.* Deficient expression of *EGR4* and *EGR1* has been observed in cryptorchid boys [28]. Thus, the observed decrease in *FGF* expression may explain in decreased prokineteicin2 (*PROK2)* gene expression, inducing central hypogonadotropic hypogonadism and impaired epididymal mesoderm development, which results in abnormal descent of the epididymal-testicular union.

### Müllerian-inhibiting substance and *INSL3*

In recent years, several genes have been found to be involved in the process of epididymo-testicular descent. Among the most frequently cited is *INSL3*. *Insl3* mutant mice display bilateral or rear unilateral cryptorchidism [29, 30] and these mutants show a feminized gubernaculum (scrotal anlage) with a deficient mesenchymal core. Thus, *Insl3* appears to have some role in the gubernacular (scrotal anlage) swelling reaction [31]. Furthermore, these mutants are claimed to have a normal epididymal development [30] which contrasts with our observation of arrested epididymal development [32]. *Insl3* mutant mouse epididymis lacks smooth musculature because of defective α-smooth muscle actin, which results in a high intraabdominal undescended position of the epididymo-testicular unit [32].

Of interest, Emmen et al. [33] reported that *Insl3* is not essential for Wolffian duct growth, and that the Müllerian-inhibiting substance (AMH) does not influence gubernaculum growth. Moreover, mice with mutations in AMH and the AMH receptor, and mice with intrauterine immunization against MIS show normal epididymo-testicular descent and normal scrotum development [34–36]. Although MIS has no obvious effect on epididymo-testicular descent in mice, it is still thought to affect human descent [37]. Hutson’s group investigated boys with androgen insensitivity and found all testicles localized in the inguinal region, indicating that the ‘first phase’ of descent is androgen-independent and MIS-dependent [38]. In contrast, abdominal testes were found in 86% of patients showing a complete female phenotype, whereby decreasing incidence was associated with increasing masculinization [39]. Thus, in patients with complete androgen insensitivity, the testicular position correlates with the genital phenotype and degree of androgen insensitivity [39]. Furthermore, anti-Müllerian hormone (AMH), INSL3 and INHB hormone levels did not differ between cryptorchid and control boys [40]. Of importance, in persistent Müllerian duct syndrome abnormal androgen dependent epididymal development is a common phenomenon [41, 42].

### Cryptorchidism

Cryptorchidism affects 1–3% of boys and is one of the most common endocrine diseases in childhood. The ultimate aim of all therapeutic approaches to cryptorchidism is to have both testes in the scrotum and to achieve a normal adult fertility potential.

#### The etiology of cryptorchidism

Epidemiologic studies have identified low birth weight as a factor very strongly associated with cryptorchidism, with additional evidence suggesting that maternal smoking and gestational diabetes further increase the risk [1, 43]. Barthold et al. recently completed one of the largest published case–control studies of congenital and acquired surgically treated cases, and found that the association with cryptorchidism in first degree relatives was significant for both congenital (odds ratio [OR]: 6.1; 95% CI: 2.0, 18.8) and acquired (OR: 8.7; 95% CI: 2.9, 26.5) presentations, but reported no differences in parental transmission rates [1].

Our recent data are consistent with the hypothesis that hypogonadotropic hypogonadism in cryptorchid boys with altered mini-puberty is the consequence of a profoundly altered gene expression program involving protein-coding genes and long noncoding RNAs (lncRNAs) [44]. We observed an increased abundance of long noncoding RNAs participating in epigenetic processes, including *AIRN*, *FENDRR*, *XIST*, and *HOTAIR*. Thus, the observed increase in familiar cryptorchidism may reflect an epigenetic phenomenon [44]. In our previous study we reported that the incidence of hypogonadotropic hypogonadism in isolated congenital undescended testes is as high as 70%.

**Fig. 12:**
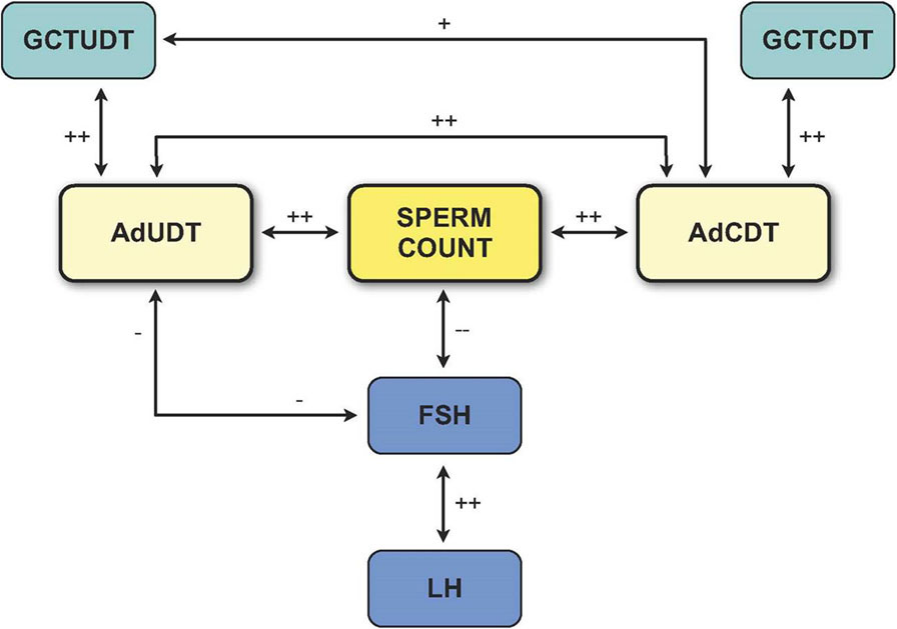
Dominant role of Ad spermatogonia in predicting fertility outcome is outlined. + + / − −; strong correlation; +/− significant correlation. Ad CDT (scrotal testis) is the best predictor of future fertility. Ad UDT (undescended testis) is a decisive factor for supporting an FSH negative feedback mechanism. GCT UDT (total germ cell count in undescended testis) and GCT CDT (total germ cell count in scrotal testis) have no direct influence either on the sperm count or on the plasma FSH level

Evidence of a relative postpubertal gonadotropin deficiency became even clearer when LH plasma values were correlated with the presence of Ad spermatogonia [45]. In addition, in our long term prospective follow-up study, hormonal analyses confirmed previous observations of an inverse correlation between follicle-stimulating hormone (FSH) levels and sperm count [45]. Gonadotropin levels, however, were more highly correlated with the presence or absence of Ad spermatogonia than with the number of undescended testes (Fig. 12) [45]. Thus, with their reliance on testicular position without support from histological findings, it is not surprising that Suomi et al. could not confirm hypogonadotropic hypogonadism in the cryptorchid boys in their population [46]. The patients with the greatest impairment of mini-puberty and who completely lacked gonocytes to Ad spermatogonia transformation were those with the most severe infertility. Consequently, the relative FSH and LH deficiencies observed in most of cryptorchid patients further support the hypothesis that hypogonadotropic hypogonadism is the main etiologic factor in cryptorchidism [45, 47–49].

The vast majority of data available support the conclusion that in many boys with undescended testes the response of Leydig cells to human chorionic gonadotropin (hCG) is diminished as compared to normal boys (for review, see [50]). Except for a blunted testosterone response to human chorionic gonadotropin, there is no evidence of altered steroidogenesis in cryptorchid testes prior to puberty [50]. Pretreatment of cryptorchid boys with hCG cancelled out the differences in their response to a stimulation test as compared to a control population [51]. Thus, the cause of the lower testosterone response seems to be at the pituitary or hypothalamic level, and may be a result of insufficient Leydig cell stimulation. Numerous LH-RH tests have demonstrated abnormally low LH response in cryptorchid boys [47–55]. Therefore, these results do not support the hypothesis that mild Leydig cell dysfunction resulting from the end organ dysgenesis is the etiologic factor for cryptorchidism, as postulated by Toppari et al. [56].

#### A tale of two testes: The bilateral impact of a unilateral disease

In 1987, Schindler et al. analyzing 495 testicular biopsies from cryptorchid boys and found that the scrotal testes in unilateral cryptorchidism showed germ cell loss in 30.1% [57]. In the remaining scrotal testes, the germ cell counts were in the low normal range with a significantly lower mean than that seen in scrotal testes associated with unilateral anorchia. Control biopsies were performed several months or years after orchidopexy in 18 boys with unilateral and in 24 boys with bilateral cryptorchidism. Orchidopexy did not improve the number of germ cells in either originally cryptorchid or in scrotal testes; the only postoperative change was an increase in tubular diameter [57]. Furthermore, Nistal et al. reported that the benefits of orchidopexy alone were very limited thus supporting the concept of congenital dysgenesis, not only of cryptorchid testis, but also of the contralateral gonad [58]. The fact that unilateral cryptorchid males develop azoospermia 25 times more often than the control population supports the concept that unilateral cryptorchidism is in fact a bilateral disease [59]. Finally, in boys with unilateral cryptorchidism, the testicular pathology caused by hormonal imbalance was bilateral; a total of 71% of scrotal testes had a reduced number of germ cells and 75% showed impaired gonocytes-to-Ad spermatogonia transformation [45].

When the incidence of paternity is analyzed, however, a distinct contrast to the above described histological findings becomes obvious. According to Lee et al., the percentage of unilateral cryptorchid men who achieve paternity does not differ from the unaffected population [60]. It should be noted that the study did not analyze testicular biopsies and depends solely on the fact that patients were orchidopexied and therefore considered to be cryptorchid [60]. However, the inevitable inclusions of low as well as intermediate and a high infertility risk patients and those misdiagnosed cases of retractile testes distorted the results. Therefore, the infertility outcome in cryptorchid boys should be assessed only after histological analysis of their testicular tissue, preferably of both testicles.

#### Histology of cryptorchid testis

It is generally believed that cryptorchidism represents heterogeneous group of disorders, which may explain controversies that persists about the long term consequences and treatment [1, 4]. From a histological point of view no such heterogeneity exists. The cryptorchid testis has a typical histology characterized by the depletion of germ cells resulting from impaired maturation of gonocytes accompanied by interstitial fibrosis, developmental arrest of Sertoli cells, and a diminished number of Sf (fetal) and Sb (polarized) Sertoli cells [61–67]. A hallmark of cryptorchid testis is a pronounced Leydig cell atrophy which also supports endocrinopathy as etiological factor in cryptorchidism [64, 67].

Thus, according to the histological picture the etiology of cryptorchidism is not in 80–90% of cases, unknown, as postulated by Virtanen and Toppari [68], but in contrast, 70% of cryptorchid testes show signs of hypogonadotropic hypogonadism with variable degree of impaired transition of gonocytes into Ad spermatogonia and distinct Leydig cell atrophy [45, 62, 66].

#### Mini-puberty and germ cells

During mini-puberty, which occurs between 30 and 90 days of postnatal life in male infants, the substantial increase in GnRH secretion induces gonadotropin and testosterone production [69]. Testicular changes during the mini-puberty are further characterized by a small peak in testicular weight [70] and volume [71]. As a consequence, transformation of gonocytes into Ad spermatogonia takes place. Ad spermatogonia have a characteristic nuclear feature that distinguishes them from the other germ cells (e.g. fetal, transient, and Ap spermatogonia) [72]. Ad spermatogonia are flat, with a long oval nucleus, and are the only spermatogonia to have a region of rarefaction within the nucleus [72]. Adult dark (Ad) spermatogonia appear at three months of age and remain present in the testis for the rest of an individual’s life [64, 72]. Therefore, the transformation of gonocytes into Ad spermatogonia, either directly or through intermediate stages, is not simply another step in a succession of developmental stages, but a major transformation. It represents the switch from a fetal reservoir of stem cells (gonocytes) to an adult reservoir of stem cells (Ad spermatogonia) from which all future germ cells are generated.

Based on results from our previous work we know that development of Ad spermatogonia is dependent on LH and testosterone [73]. Cryptorchid infants have an impaired testosterone increase during mini-puberty [47, 49, 51, 74]. Furthermore, cryptorchid boys lacking Ad spermatogonia have low basal and stimulated gonadotropin plasma values that are compatible with those found in cases of hypogonadotropic hypogonadism [75]. If transformation during infancy of gonocytes into Ad spermatogonia fails, infertility is inevitable [45, 76]. Kim et al. analyzed the histology of testicular biopsies from patients with bilateral cryptorchidism and correlated it with post pubertal semen analysis, thereby confirming the prognostic importance of Ad spermatogonia for fertility [77].

#### Mini-puberty and Sertoli cells

Once spermatogenesis is established, the Sertoli cells hold the developing germ cells either in the spaces between adjacent pairs of cells, or in crypts in their luminal surface. Each Sertoli cell can sustain only a limited number of germ cells; therefore the number of Sertoli cells per testis determines the highest possible sperm production. The number of Sertoli cells in cryptorchid testes has been only sporadically studied [61, 78–80]. Cortes et al. [78, 79] found that a significant increase in the number of Sertoli cells occurs during the first three months of life. We reported, however, that during the first four months of life no difference could be found between the number of Sertoli cells in cryptorchid patients and patients with spontaneously descended testis (22.38 ± 1.01 compared to 23.53 ± 1.98) [81]. As age increases (5–12 months), this difference becomes more pronounced (23.20 ± 1.41, with cryptorchidism compared to 26.20 ± 1.40, in controls; *p* = 0.001) [81]. Thus, the insufficient increase in the number of Sertoli cells is in accordance with recently observed significantly lower testosterone, AMH and inhibin B plasma values in cryptorchid boys [82]. Therefore, an early postnatal increase in inhibin B is presumably due to the activation of the hypothalamic-pituitary testicular axis [83] and mirrors the proliferating activity of Sertoli cells. Of interest, the early postnatal rise of inhibin B is better correlated with LH and testosterone than with FSH [84, 85], raising the possibility that Sertoli cell proliferation in neonatal life depends more on LH/testosterone than on FSH [86].

### Current treatment options

The greatest challenge in any clinical procedure for undescended testes is to exclude the retractile testis, for which no treatment is required except categorical reassurance. Inadequate examination has certainly fooled many doctors and even experienced surgeons many times [87]. More than half of the patients sent for treatment fall into this category [87]. If the retractile (but otherwise normal) testis is ‘treated’, the ‘outcome’ is obviously bound to be good. It is vital to exclude such cases from the procedures [87, 88].

### Should surgery alone remain the best treatment recommended in guidelines?

A recent equivocal summary of research on the physiology of testicular descent has resulted in a Nordic consensus article [89]. The Nordic consensus on the treatment of undescended testis relies greatly on observations reported by Ritzén et al. and Kollin et al. [90–92]. The key message was that the observed germ cell loss was caused solely by the age at the treatment. “Support” for early surgery was several years later given by Feyles et al. in their long-term fertility study [93]. Total sperm count in boys having orchidopexy before one year of age (52.3 ± 14.3 million/mL vs.30.4 ± 23.5 million/mL) was not significantly different between two groups, while percentage of those with normal sperm count according to WHO standards was higher in male having had surgery before first year of life (96% vs 75%; *p* < 0.042) [93]. Of note, and this is important, the number of patients who developed azoospermia or severe oligospermia did not differ between patients who had surgery before and after the first year of age (*p* = 0.39, Fisher’s Exact test) [94]. Indeed, in Feyles et al. study, severe infertility and azoospermia developed, irrespective of the age of treatment [94]. The key conclusions from Kollin et al. study are misleading for several reasons:

**First,** despite technical adequacy of histological tissue preparations, the responsible histologists clearly did not differentiate between gonocytes and spermatogonia in the germ cell population. This differentiation is important, because between nine months and three years in the normal testis, 40% of germ cells are lost due to the translation of gonocytes into spermatogonia, which is not induced by the cryptorchid position [6, 64, 94]. Thus, spermatogonia count and not the total cell count between two groups should be compared. **Second,** in the last 30 years, it has been convincingly shown that three-year-old, unilateral cryptorchid boys have an average germ cell count of 0.32 germ cells per tubular cross-section [64, 94, 95]. Of note, in this study, 3-year-old cryptorchid boys had an average germ cell count that was 320% lower than expected. This suggested either selection bias or invalid histological analysis.

**Third,** the occurrence of Ad spermatogonia indicates completion of mini-puberty, and is an excellent parameter for predicting fertility outcome in cryptorchid boys [45]. Worth noting, only a comparison between groups of cryptorchid boys that lack Ad spermatogonia in both testes with a group of cryptorchid boys that have Ad spermatogonia in both testes could confirm previously reported observations that cryptorchid boys lacking Ad spermatogonia have hypogonadotropic hypogonadism, and should be treated with LH-RHa following successful and early orchidopexy, to escape infertility [75, 96]. This comparison, despite adequate histological tissue preparation, was not performed by Kollin et al. [91]. Because of the inexperience of the histologist the analysis of testicular tissue was inadequate, which resulted in unsubstantiated and, misleading conclusions. Therefore, the postulated key message; *that orchidopexy at 9 months is more beneficial for testicular development than an operation at 3 yr. of age*, was based on incorrect histological data assessments.

**Fourth,** In contrast to the concerns raised by Nordic consensus group regarding harmful effect of hormonal treatment, the results of our study showed that hormonal treatment for undescended testis improved the histopathology of the contralateral testis without harming the germ cells [97, 98].

**Finally**, because testicular volume does not accurately predict germ cell count in patients with undescended testes, this parameter cannot be used to select patients for post-orchidopexy hormonal therapy. Therefore, testicular volume cannot replace the predictive value of testicular biopsy in the modern management of cryptorchidism and early surgery alone is an insufficient attempt to correct infertility development in at least 40–50% of cryptorchid boys. Testicular biopsy is all the more justified because it allowed detection of in situ carcinoma in 0.6% of the cryptorchid boys that were studied.

Of interest, although the Nordic consensus group rejects hormonal treatment, recently one of the authors from this group recommended hormonal treatment for cryptorchidism in cases of bilateral cryptorchidism with hypogonadotropic hypogonadism [99].

### Forgotten history and ignored observations

The main reason for not recommending hormonal treatment of the undescended epididymo-testicular union is supposedly that the success rate of this treatment is as low as 20% [90, 92]. This statement is misleading because it does not consider the distribution of the positions of the epididymo-testicular unit before treatment. Of importance, not performing three arm randomized placebo controlled studies could falsify success results because of the inclusion of retractile testes. Furthermore, in most of the studies the long follow-up period was short.

An approach other than a three-armed study was performed by Höcht in 1995. This study was randomized with LH-RH or surgery groups including 60 cryptorchid boys aged 2 to 9 years [100, 101]. All patients randomized for the surgery treatment alone had histological changes compatible with cryptorchidism, so it is most likely that only undescended and not also retractile testes were treated. LH-RH treatment was successful in 59% of the patients. Ten years after the treatment 52% of testes remained descended which is in sharp contrast to the 20% re-ascent claimed by Nordic consensus group [92]. Thus, LH-RH treatment is effective in achieving permanent descent of true cryptorchid testes [101]. The highest success was achieved when testes were localized pre-scrotal (Table 1).

**Table 1:** Descent rate of epididymo-testicular unit from pre-scrotal position treated with LH-RH

Autor(s)	Year	n	% success
1. De Muinck Keizer-Schrama et al. [116]	1986	6/9	66
2. Borkenstein and Zobel [117]	1987	5/9	55
3. Hagberg and Westphal [118]	1987	8/17	47
4. Höcht [101]	1987	3/4	75
5. Bica and Hadziselimovic [17, 119]	1993	6/11	54.5
		28/50	56

Waldschmidt et al. described two independent long term follow-up studies of seven and five years after LH-RH treatment, respectively. Seven years post-treatment 50.5% of the testes were in a scrotal position, while in the second study with a five year follow-up, 67% of the cryptorchid testes were in the scrotum [102]. Of importance, in a uniquely designed study, assigning the patients with cryptorchidism randomly into three-arm groups, GnRH treatment induced complete epididymo-testicular descent only in the treated group [17]. Furthermore, boys treated with buserelin had the highest number and the best maturation index of the germ cells [17].

In conclusion, hormonal pretreatment should remain the first therapeutic choice because it avoids resorting to surgery; In addition; it has no adverse effect on fertility and, in unsuccessful cases, facilitates orchidopexy and considerably helps reduce the incidence of postsurgical testicular atrophy.

### Combining classical physiological information and the output of cutting-edge genomics data into a complete picture

#### Temperature or transposon de-regulation

It is widely accepted that exposure of the cryptorchid testis to abnormally high temperatures induces germ cell loss and infertility [103]. However, the temperature effect cannot explain testicular development of boys with deficiencies in 5-alpha reductase with bilateral cryptorchidism. These boys exhibit normal spermatogonia numbers and normal germ cell differentiation during the entire prepubertal period. Those findings are in sharp contrast to observations in boys with isolated bilateral cryptorchidism having identical testicular malpositioning [104]. Because the testicular positions in these two groups of boys are identical, their testes would be expected to have experienced the same temperature exposure. Therefore, if temperature was the only explanation for a massive germ cell loss in isolated bilateral cryptorchidism, the two groups should have exhibited identical germ cell loss. This, however, this was not the case [104]. Thus, at the level of spermatogonia, temperature appears to have only a negligible effect in contrast to the late germ cell developmental stages (adult testis).

The decreased germ cell count found in this group of boys could be the result of uncontrolled transposon activity inducing genomic instability and germ cell death. When we performed a genome-wide RNA profiling analysis, we found in our first study that genes important for transposon silencing were not expressed in the high infertility risk group of cryptorchid boys but only in the low infertility risk and control groups [105].

Intact mini-puberty appears to be essential for the development of the endogenous defense system mediated by transposon silencing [105, 106]. Spermatogonia contain processing bodies that harbor P-element-induced wimpy testis (Piwi) proteins. Piwi proteins are associated specifically with Piwi-interacting RNAs to silence transposable DNA elements. Loss-of-function mutations in the Piwi pathway lead to de-repression of transposable elements, resulting in azoospermia and infertility [106]. Furthermore, deletion of gametocyte-specific factor 1 *(GTSF1),* a protein involved in Piwi-mediated transcriptional repression, causes male-specific sterility and de-repression of LINE-1 (L1) retrotransposons. Seven members of the Tudor gene family, and three members of the DEAD-box RNA helicase family were found in our second study to show significantly lower RNA signals in the high-infertility-risk group. In the immune-histochemical analysis, patients from the low-infertility-risk group showed coherently stronger staining for GTSF1 and PIWIL4 proteins and weaker staining for the L1 transposon when compared to the high-infertility-risk samples. These findings provide the first evidence consistent with the idea that infertility in cryptorchidism is a consequence of alterations in the Piwi pathway and the de-repression of transposon induced by the impaired testosterone function during mini-puberty [106].

#### Idiopathic central hypogonadotropic hypogonadism

Recent observation of lower plasma LH levels in cryptorchid boys with the most pronounced testicular pathology and impaired gonocytes transformation [94], confirms previous findings that this group of cryptorchid boys suffers hypogonadotropic hypogonadism [75]. The estimated incidence of defective mini-puberty in boys with cryptorchidism could be as high as 70% [45, 107]. Using whole genome RNA profiling of testicular biopsies by DNA strand-specific RNA sequencing we found multiple differences in gene expression between the high and low infertility risk groups, confirming the importance of an intact hypothalamus-pituitary testicular axis for fertility development. [28, 44, 108, 109] Furthermore, we observed decreased *PROK2, CHD7, FGFR1,* and *SPRY4* expression in the high infertility risk group of cryptorchid boys [104]. In particular, *EGR4 and PITX1*, which are involved in regulating the secretion of luteinizing hormone, were virtually not expressed [108].

Our RNA profiling data strongly support the theory that in the high infertility risk group of cryptorchid boys insufficient *PROK2*/*CHD7*/*FGFR1*/*SPRY4* gene expression, together with observed deficient *EGR4* and *PITX1* signaling, induce deficient LH secretion, which results in impaired mini-puberty [28, 44, 105, 108, 109]. Of interest is that in bilateral cryptorchid infants with hypogonadotropic hypogonadism having Kallmann-, Charge syndrome or panhypopituitarism, displaying undetectable LH and testosterone plasma level. Daily subcutaneous injections of the recombinant LH 75 + FSH 150 IU for a period of three months rescued mini-puberty repairing micropenis and cryptorchidism (all testes descended). [110] The treatment also induced high-normal activation of Leydig and Sertoli cells as well as normal LH, FSH, INHB, AMH and testosterone levels [110]. Identical hormonal results and 75% successfully descended testes were achieved in bilateral cryptorchid boys with congenital hypogonadotropic hypogonadism using subcutaneous LH and FSH infusions [111].

#### GnRH treatment to rescue fertility

In a substantial number of cryptorchid males, early and apparently successful orchidopexy does not improve fertility, because it does not cure the underlying pathophysiological cause, which is the impaired transformation of gonocytes into Ad spermatogonia [28, 45]. It is important to realize that more than half of the patients presenting with unilateral cryptorchidism, and the majority of those presenting with bilateral cryptorchidism, have an abnormal semen analysis, which indicates that unilateral cryptorchidism is in fact a bilateral disease and therefore a serious andrological problem [59]. Despite timely and successful surgery, 32% of patients with bilateral and 10% with unilateral cryptorchidism will develop azoospermia [59].

Thus, cryptorchidism represents the most common cause of non-obstructive azoospermia in men. GnRH therapy is a worthwhile solution for this andrological problem. Initial results of GnRHa treatment on alternate days over a period of six months showed that this treatment did not inhibit gonadotropin secretion, while LH levels increased and atrophic juvenile Leydig cells regenerated toward the end of the treatment [112]. Thus, the treatment increased the number of germ and Leydig cells [97, 112]. Cryptorchid boys of a median age of eight years, who were treated with a gonadotropin-releasing hormone agonist, showed after puberty improved sperm concentrations when compared to an untreated control group [113]. Worth noting, long term follow-up in high infertility risk group of cryptorchid boys treated before the age of six showed normal sperm concentrations in 86% of cases [114]. This results strongly contrasts with those of the ‘surgery only’ group in which not a single patient had a normal semen analysis and 20% suffered from azoospermia [114].

Finally, a recent prospective randomized study designed for boys with isolated bilateral cryptorchidism without Ad spermatogonia in the testicular biopsy were randomly divided into two groups. One group underwent the second orchidopexy without any hormonal treatment and the second group received intranasal LH-RHa therapy for six months followed by a second orchidopexy. In contrast to the boys treated with surgery alone, all patients from the LH-RHa treated group completed the transition of gonocytes into Ad spermatogonia (*p* = 0.008). Thus, treatment with LH-RH agonist (buserelin) is beneficial and should be considered in an attempt to correct impaired mini-puberty to improve the fertility potential in cryptorchid boys [115]. At the molecular level, GnRH induces a significant transcriptional response, including protein coding genes involved in pituitary development, the hypothalamic-pituitary-gonadal axis, and testosterone synthesis. Expression patterns of prepubertal germ cells indicate that genes involved in meiosis and post meiotic germ cell development are already up-regulated before puberty [28, 108]. The observed differential gene expression profiles of gonocytes and spermatogonia markers, especially *DMRTC2*, *PAX7*, *T* (Brachyury) and *TERT*, highlight their importance for the development of Ad spermatogonia with specific functions in self-renewal and differentiation [109]. Furthermore, we could show that in GnRH treated high infertility risk patients, the development of Ad spermatogonia is rescued through the activation of alternative genes. We suggest that GnRH -induced elevated testosterone and LH levels reconstitute self-renewal properties of the Ad spermatogonial stem cells and help prepare them for commitment to differentiation, by inducing retinoic acid responsive genes such as *NRG1*, *NRG3* and *PAX7* [109]. Together with our earlier observations on the level of the hypothalamic-pituitary-gonadal-axis of differentially expressed genes in high infertility risk patients we suggest that *EGR4* and *PITX1* controlled by *PROK2/CHD7/FGFR1/SPRY4* gene expression is responsible for LH deficiency, which in turn affects germ cell transitional effectors *FGFR3*, *FGF9*, *NANOS2*, *NANOS3*, *SOHLH1* and *SOHLH2*. Upon GnRHa treatment, however, alternative pathways are activated including the LH-secretion orchestrating factors *EGR2*, *EGR3*, *TAC1*, *TAC3*, *PROP1* and *LEP,* and the gonocytes-to-Ad spermatogonia transition effectors *DMRTC2*, *T*, *PAX7*, *TERT*, *NRG1*, *NRG3*, *RBMY1B*, *RBMY1E* and *RBMY1J* [109] (Table 2).

**Table 2:** Pathophysiology of cryptorchidism induced infertility. Treatment with GnRH permanently stimulates alternative pathway to induce transition of gonocytes and undifferentiated spermatogonia into Ad spermatogonia

FGFR1	FGFR1 **↓**	FGFR1 ↓
↓	↓	↓
PROK2	PROK2 **↓**	PROK2 ↓
↓	↓	│ ← **GnRH**
GnRH	GnRH **↓**	EGR2 / EGR3 **↑**
↓	↓	↓
EGR4 / PITX1	EGR4 /PITX1 **↓**	LH **↑**
↓	│	↓
LH	LH **↓**	HSD17 B2 **↑**
↓	│	CYP19A1 **↑**
Testosterone	Testosterone **↓**	Testosterone **↑**
↓	↓	↓
T-brachyury,POU2F2	T-brachyury **↓**	POU2F2 **↑**T-brachyury **↑**
↓	↓	↓
Ad	No Ad	Ad
↓	↓	↓
Fertility	Infertility	Fertility

## Conclusion

Five main conclusions are possible based on the available evidence to date. **First,** hypogonadotropic hypogonadism is the most common cause of cryptorchidism. Molecular observations support a crucial role for PROK2 in the pathophysiology of cryptorchidism with impaired *PROK2/CHD7/FGFR1/SPRY4* gene expression inducing LH deficiency as controlled by the regulators *EGR4* and *PITX1.* **Second**, unilateral cryptorchidism is a bilateral disease and a serious andrological problem. **Third,** hormonal treatment successfully induces epididymo–testicular descent (Table 3). **Fourth,** the most severe associated infertility is seen in men with a history of cryptorchidism who had the greatest impairments in mini-puberty and completely lacked gonocytes translation into Ad spermatogonia in both testes (Table 3). **Finally**, when patients at increased risk of infertility (those lacking Ad spermatogonia) received treatment with GnRH, normal semen concentration analyses were observed in 86% of cases. Omitting GnRH treatment, one third of men with a history of defective mini-puberty will develop azoospermia despite successful surgery.

**Table 3:** Basel concept of cryptorchidism treatment

1.	LH-RH 1.2 mg/ day for 28 days;
	If no or partial success:
2.	500 IU hCG / week for 3 weeks;
	If no descent:
3.	Orchidopexy and bilateral biopsy;
	If no bilateral Ad spermatogonia:
4.	LH-RH 10 μg on alternate day for six months.

*LH-RH* Luteinizing hormone-releasing hormone (GnRH), hCG: human chorionic gonadotropin

## Abbreviations

AIRN: Antisense of IGF2R Non-Protein Coding RNA
AMH: Anti- Müllerian hormone
*CHD7*: *Chromodomain Helicase DNA Binding Protein 7*
*CYP19A1*: Cytochrome P450 Family 19 Subfamily A Member1
DMRTC2: DMRT Like Family C2
EGR1: Early growth response 1
EGR3: Early growth response 3
EGR4: Early growth response 4
FENDRR: FOXF1 Adjacent Non-Coding Developmental Regulatory RNA
FGF: Fibroblast growth factor
FGFR1: Fibroblast growth factor receptor 1
FSH: Follicle-stimulating hormone
GnRH: Gonadotropin-releasing hormone
GnRHa: *Gonadotropin-releasing hormone agonist*
GTSF1: Gametocyte-specific factor 1
hCG: Human chorionic gonadotropin
HOTAIR: HOX Transcript Antisense RNA
HSD17B2: Hydroxysteroid 17-Beta Dehydrogenase 2
INSL3: Insulin-like factor3
IU: International unit
LEP: Leptin
LH: Luteinizing hormone
LH-RH: Luteinizing hormone-releasing hormone
LH-RHa: Luteinizing hormone-releasing hormone analogue
lncRNAs: *Long noncoding RNAs*
NANOS2: Nanos C2HC-Type Zinc Finger 2
NRG1: Neuregulin 1
OR: Odds ratio
PAX7: Paired Box 7
PITX1: Paired like homeodomain 1
PIWI 1: P-element-induced wimpy; Piwi Like RNA-Mediated Gene Silencing 1
*POU2F2*: *POU Class 2 Homeobox 2*
PROK2: prokineticin 2
PROP1: PROP Paired-Like Homeobox 1
*RAF1*: *Raf-1 Proto-oncogene, serine/threonine kinase*
*RBMY1B*: *RNA Binding Motif Protein, Y-Linked, Family 1, Member B*
Sb: Polarized sertoli cells
Sf: Fetal sertoli cells
*SOHLH1*: *Spermatogenesis and oogenesis specific basic helix-loop-helix 1*
SOS1: SOS Ras/Rac guanine nucleotide exchange factor 1
SPRY4: Sprouty RTK signaling antagonist 4
T: T Brachyury transcription factor
TAC1: Tachykinin precursor 1
TERT: Telomerase reverse transcriptase
WHO: World Health Organization
XIST: X inactive specific transcript (Non-Protein Coding)
